# Oesophageal and gastric varices: historical aspects, classification and grading: everything in one place

**DOI:** 10.1093/gastro/gow018

**Published:** 2016-06-19

**Authors:** Cyriac Abby Philips, Amrish Sahney

**Affiliations:** Department of Hepatology, Institute of Liver and Biliary Sciences, New Delhi, Delhi, India

**Keywords:** oesophageal varices, gastric varices, classification, portal hypertension

## Abstract

Variceal disease and its management are of the utmost importance in the treatment of portal hypertension. Current guidelines are universal for management of variceal disease in portal hypertension. Classification and grading systems are numerous and differ according to geographical location. In this exhaustive review, the historical aspects of variceal disease, its classification and the grading systems in use are discussed, with self-explanatory tables and timelines. A better and clear understanding of the evolution of portal hypertension and variceal disease is provided.

## History of portal hypertension and variceal disease

Before the role of portal hypertension in the development of variceal disease was understood, Nikolai Eck had in 1877 already created porto-caval shunts in animals to try to treat ‘mechanical ascites’. The ‘Eck fistula’ proved successful in eight dogs, seven of which died during the immediate postoperative period, while one survived for 2.5 months before escaping from the laboratory. The safety of diverting portal blood directly into the systemic circulation was confirmed [1]. The mapping of the portal venous system—and with it the story of oesophageal varices—began with Vesalius in 1543 [2], with Morgagni describing “a portal hypertensive bleeding” two centuries later [3]. In 1841, Raciborski was the first to discover that collaterals form between the systemic and portal circulations through the abdominal wall, short gastric and haemorrhoidal veins, with Sappey describing oesophageal varices a decade later [4, 5]. In 1903 Vidal, successfully established an Eck fistula in a man with ascites 26 years after the procedure was first described in animals [6].

Understanding of portal hypertension leading to variceal development improved slowly up to 1928, when Wolf first demonstrated, in two patients, the occurrence of oesophageal varices on thin barium Roentgenograms as small dilated luminal structures [7]. This was followed by case reports and series by Berg (1931), Hjelm (1931), Kirklin (1931), Beutel (1932) and Oppenheimer (1937), demonstrating the presence of oesophageal varices on barium studies. In 1931, Schatzki published his observations—the first on gastric varices—on Roentgenograms of five patients, followed by 45 further patients with oesophageal and gastric varices in 1933. The first description of varices was “dilated veins that bulge into the lumen, producing uneven worm like surface of the inside of oesophagus” [8]. Gilbert and Villaret coined the term ‘portal hypertension’ in 1906 [9]. Thereafter, studies in 1936 by Rousselot on patients with ‘Banti’s syndrome’ shed light on elevated portal pressures [10] and, in 1937, Thomson and colleagues confirmed these findings by portal pressure measurement during celiotomy procedures, clarifying the role of portal hypertension in the development of oesophageal varices [11].

Philip Bozzini in 1805 created a special tube, naming it the ‘Lichtleiter’ or ‘light-guiding instrument’ for examining the urinary tract. This was later renamed ‘endoscope’ by French surgeon Antoine Jean Desormeaux. Adolph Kussmaul first utilized the endoscope to examine the inside of the stomach of a living person in 1868. In 1881, Johann von Mikulicz, a Polish-Austrian surgeon, created and utilized the ‘gastroscope’, specifically for examination of the oesophagus, stomach and small intestine. The Flexible gastroscope was introduced by Rudolph Schindler in 1932 with the improved ‘gastro camera’ appearing in the 1950s [12–16]. In 1936, Crafoord and Freckner, two Swedish surgeons, first reported their use of rigid gastroscopes to visually inspect bleeding varices and stop the bleed utilizing injection sclerotherapy with quinine-urethane solution [17]. Variceal disease and its consequences have long been the commonest emergency in portal hypertension. Even to this day, 1 out of 5 patients with variceal bleeding has a mortality approaching 22% at 6 weeks. To really appreciate the treatment modalities currently in use, it is important to understand the classification systems and grading of varices.

## Classification and grading of oesophageal varices

**Figure 1.**
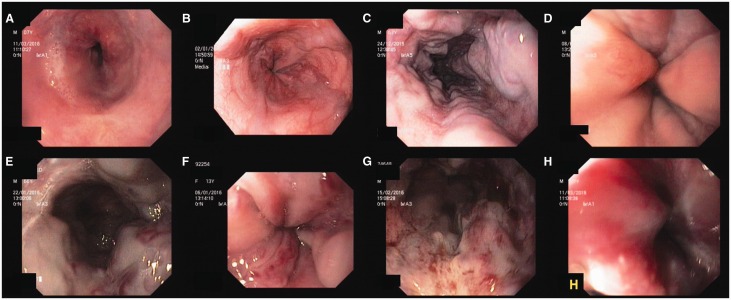
A)[Fig gow018-F2]Small oesophageal varices (grade 1, form F1); B) Small and beady oesophageal varices (grade 2, form F2); C) Large oesophageal varices (grade 3, form F3); D) Large white oesophageal varices that look like mucosal folds; E) Grade 3 oesophageal varices with red colour signs (whip-like red wale marks); F) Large oesophageal varices with red colour signs (cherry red spots and red wale marks); G) Diffuse red colour signs (red wale marks) over oesophageal varices; H) Large oesophageal varices with diffuse redness.

**Figure 2. gow018-F2:**
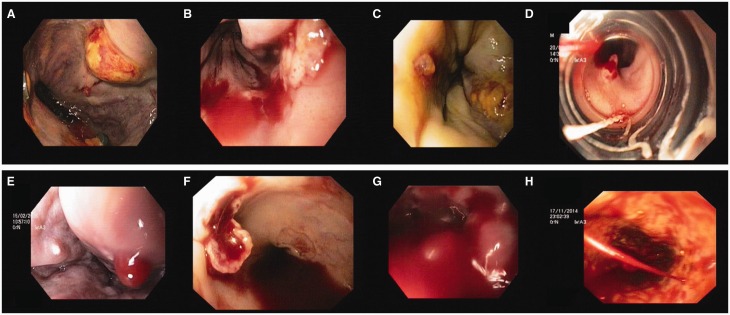
A) Grade 3 oesophageal varix with large ulcer; B) Post-band ligation status, EVL ulcer with active oozing; C) White nipple sign over an oesophageal varix—stigmata of recent haemorrhage; D) Red nipple sign over an oesophageal varix—red platelet plug, stigmata of recent haemorrhage; E) Large haemocystic spot, a high-risk sign for bleeding; F) Ruptured oesophageal varix with overlying ulcer and active ooze; G) Gushing bleed (as per Japanese Society classification) from large oesophageal varices (grade 4 classification as per Conn’s classification and Dagradi classification); H) Spurting grade 2 oesophageal varix.

(Refer to figures 1 and 2.) In 1934, Kegaries studied the portal venous system aberrancy in portal hypertension and relationships of varices in the inferior part of the oesophagus. Butler in 1951 provided a thorough account of the microvascular portal venous anatomy and, using anatomical specimens, demonstrated connections between the subepithelial and submucosal venous plexuses of the lower oesophagus [18, 19]. Accordingly the venous system of the oesophagus was described as consisting of:

- intrinsic veins, including sub-epithelial plexus, in the lamina propria, sub-mucosal plexus of veins and perforators that join the two plexuses, draining eventually into extrinsic veins
- extrinsic veins (about 20 in number formed by perforating venous groups)
- veins that accompany vagal nerves running in the adventitial wall of the oesophagus.

The group of sub-epithelial veins is most important, in that these are heavily predominant in the distal oesophagus, running longitudinally. These longitudinal veins terminate directly in the deep sub-glandular veins of the upper gastric wall, favouring a superficial location and easy bleeding from one single bleeding point [20,21]. During portal hypertension, significant collateral circulation develops: increased blood flow from the proper oesophagus and bronchial vessels—and resistance to flow in the oesophageal muscularis layer—contribute to an increase in pressure, leading to the dilatation and dislocation of veins, forming varices. When the networks of sub-epithelial and submucosal plexus veins dilate, these are referred to as ‘intra-epithelial microvarices’ and are thought to contribute to point bleeding in variceal rupture [22–24].

For the purpose of describing variceal anatomy, the oesophagus is divided into: -

- the palisade zone: as described in detail by Kegaries in 1934, this zone begins at the gastro-oesophageal junction and extends superiorly for 4–5 cm, at which point blood that mainly comes from the superior vena cava is in contact with portal blood, as described by De Carvalho in 1966. In this zone, sudare-like veins exist (known as Group 1 when seen in persons without portal hypertension or Group 2 when seen in patients with well developed portal hypertension consisting of high vascularity with interspersed dilated connecting channels). Hashizume called the former type ‘pallisading’ and the latter ‘bar type’. In the bar type, vertically oriented vessels connect to 3–4 tortuous dilated vessels and are significant in causing bleeding related to portal hypertension.
- the truncal zone, consisting of 3–4 dilated and tortuous vessels (an area where most commonly varices form).
- the transitional zone, in which both these types of veins enter.

In 1987, utilizing radiology, morphometry and corrosion casting, Vianna *et al*.** elegantly described oesophageal varices in relation to normal anatomy and defined four different drainage zones: (i) the gastric, with longitudinal venous distribution, (ii) the palisade, comprising groups of parallel vessels within the lamina propria with high resistance bidirectional flow (watershed region between azygous and portal system), (iii) the perforating zone with treble clef-shaped veins draining into extrinsic veins and (iv) the truncal zone, with 4–5 deep-lying descending veins. In the perforating zone, perforators run through the muscle wall, connecting submucosal and paraesophageal venous plexuses, which are azygous system tributaries. In portal hypertension, the dilated perforators become incompetent and allow retrograde flow of blood from the extrinsic (paraesophageal) to the intrinsic (submucosal) veins which, in association with turbulence of flow related pressure changes during respiration, coughing etc., lead to dilatation and formation of varices, also making this area the critical area for variceal rupture [25].

In 1955, Brick and Palmer first classified oesophageal varices as [26]: -

- those in association with portal hypertension (intrahepatic and extrahepatic causes)
- those associated with portal hypertension without any clinical or anatomical causes delineated
- those associated with hypertension in the superior vena cava
- primary varices (true varices) limited to the upper part of the oesophagus
- idiopathic varices without portal or superior vena caval hypertension.

The first attempt at grading oesophageal varices by way of rigid oesophagoscopy was also made by Brick and Palmer in 1964 [27]. They graded varices as mild (<3 mm in diameter), moderate (3–6 mm) and severe (>6 mm) on direct visualization. Initially a bluish colour was supposedly one of the most important characteristics for distinguishing varices from mucosal folds, but its absence meant little. A more reliable factor was found to be the tortuous course (in higher grade varices) or persistence of mucosal elevation during respiration (for lower grades). Gastrointestinal endoscopy in portal hypertension is utilized to confirm the presence of variceal disease, to assess risk of complications secondary to variceal disease and to actively manage acute variceal bleeding.

A detailed attempt at the classification of varices was that of Dagradi (1966, modified in 1972; Table 1A), who also, for the first time, identified the red colour sign and cherry red spot (CRS) [28].

The Conn classification of oesophageal varices came into being in 1967 (Table 1B) [29]. As with earlier descriptions, this classification also dealt mostly with the presence and size of varices without mucosal descriptions. To improve the detection of varices, he proposed the use of red filters and/or colour photography. Conn also proposed diagnosing variceal disease using an ammonia clearance test and without need for upper gastrointestinal endoscopy. In his series, arterial blood ammonia levels above 250 μm/100mL was in agreed in 70% with other methodologies for detection of varies in cirrhotics. This was one of the earliest attempts at non-invasive diagnosis of varices, a topic that is outside the scope of this review. Further work on the evolution of grading of varices came from Japan, with Sannohe *et al*.** describing varices with an emphasis on colour and pointing out the importance of the ‘red plug’ as a high-risk sign [30]; Kumagai *et al*.** described the shape and surface of the varices in their classification [31], while Endo *et al*.** described important high-risk signs, such as ‘varix on varices’ [32].

**Table 1.** Classification of oesophageal varices

A. Dagradi classification	B. Conn’s classification
**Grade 1:** Varices are blue or red in colour and are brought out by compression of the oesophageal wall with the tip of the oesophagoscope. Usually linear, sometimes sigmoid shaped, and less than 2 mm in diameter. **Grade 2:** Varices are bluish in colour, 2–3 mm in diameter, mildly tortuous or straight, and are elevated above the surface or the relaxed oesophagus **Grade 3:** Prominently elevated bluish veins 3–4 mm in diameter are either straight or tortuous. **Grade 4: **Tortuous bluish varices more than 4 mm in diameter, which completely surround the oesophageal lumen and almost meet in the mid-lumen; are closely packed around the wall and may or may not have a good mucosal cover. **Grade 5:** Varices are grape-like in appearance, occlude the lumen of the advancing oesophagoscope and demonstrate the presence of small, cherry-red varices overlying the large, slightly deeper lying, slate blue-grey varices (also known as ‘varices over varices’).	**Grade I:** Visible only during one phase of respiration/performance of Valsalva manoeuvre. **Grade II:** Visible during both phases of respiration. **Grade III:** 3–6 mm in diameter. **Grade IV:** >6 mm in diameter.
	**C. Paquet’s classification**
	**Grade I:** Microcapillaries located in distal oesophagus or oesophago-gastric junction. **Grade II:** One or two small varices located in the distal oesophagus. **Grade III:** Medium-sized varices of any number. **Grade IV:** Large-sized varices in any part of oesophagus.
	**D. Westaby classification**
	**Grade 1:** Varices appearing as slight protrusion above mucosa, which can be depressed with insufflations. **Grade 2:** Varices occupying <50% of the lumen. **Grade 3:** Varices occupying >50% of the lumen and which are very close to each other with confluent appearance.
**E. Soehendra classification**	
**Grade I**: Mild dilatation; diameter <2 mm; barely rising above relaxed oesophagus; more marked in head down position. **Grade II**: Moderate dilatation; tortuous; diameter 3–4 mm; limited to the lower part of the oesophagus. **Grade III**: Total dilatation; taut; diameter >4 mm; thin-walled; varices upon varices; in gastric fundus. **Grade IV:** Total dilatation; taut; occupy the entire oesophagus; frequent presence of gastric or duodenal varices.	
	**F. Calès classification**
	**Grade 1:** Varices flattened by insufflations. **Grade 2:** Varices not flattened by insufflations and separated by areas of normal mucosa. **Grade 3:** Confluent oesophageal varices not flattened by insufflations.

It was not until 1980 that the Japanese Society for Portal Hypertension (JSPH) created their General Rules unifying the descriptions of endoscopic findings of variceal disease, providing a detailed macroscopic evaluation and leading to one of the most important steps in classification and grading of variceal disease [33]. This became the first step in streamlining documentary evidence of variceal disease. This was subsequently modified with minor changes in 1991 (Table 2A); this system consisted of six main categories: the four main categories of the 1980 classification were location (L), form (F), fundamental colour (C) and red colour sign (RC), to which, in the revised classification, were added bleeding sign (B) and mucosal findings. Another change was that the separate groupings of gastric varices in the earlier classification were compiled into a singular classification under the heading ‘oesophago-gastric’ varices. Also, gastric varices were described in the same way as oesophageal varices. Colour descriptions were either white or blue, with white varices resembling large folds of oesophageal mucosa and blue varices that were cyanotic-looking and distended by blood, with the overlying oesophageal mucosa appearing very thin. A blue varix, considered dangerous in the absence of classical red colour signs (RC) is also characterized by its shiny, glossy appearance (like an overinflated balloon). Red wale markings (RWM) are longitudinal, whip-like or wormy red markings; cherry red spots are small red spots and haematocystic spots are crimson-coloured, large, blood-filled projections or blister-like lesions (usually single, over a large tortuous varix) seen on the mucosal surface of the varices. Red colour is classified depending on its density of distribution: small in number/localized uses the symbol (+), while large in number, diffuse or circumferential uses (+++), with (++) falling in between. The notable finding of diffuse redness (DR), which was previously included in the 1980 classification, was deleted from the 1991 revision in view of the difficulty of distinctively defining this entity. In the previous classification, the only mucosal finding was oesophagitis, which was extended to erosion, ulcer or scarring in the current description.

**Table 2.** The Japanese Society for Portal Hypertension, Beppu, and Snady and Feinman classification and documentation of oesophageal and oesophageo-gastric varices

A. JSPH classification	B. Beppu classification	C. Snady and Feinman’s classification
Location (L) • Locus superior (Ls) • Locus medialis (Lm) • Locus inferior (Li) • Locus gastrica (Lg) • Gastric varices adjacent to the cardiac orifice • (Lg-c) • Gastric varices distant from the cardiac orifice • (Lg-f) • Gastric varices extending from cardiac orifice to fornix (Lg-cf)	Location (L) • Locus inferior (Li) [+0.1675] • Locus medialis (Lm) [−0.0045] • Locus superior (Ls) [−0.0319]	Location (L) • Varices located within the lower one-third of the oesophagus (distal 6 cm) [0] • Varices located within the lower two-thirds of the oesophagus below the tracheal bifurcation [1] • Varices extending above the tracheal bifurcation to the cricopharyngeus [2]
Form (F) • Lesions assuming no varicose appearance (F0) • Straight small-calibre varices (F1) • Moderately enlarged, beady varices (F2) • Markedly enlarged, nodular, or tumour-shaped varices (F3)	Form (F) • Straight varices (F1) [+0.2622] • Enlarged tortuous varices (F2) [+0.1312] • Largest sized varices (F3) [-0.1020]	Form (F) • Small, straight varices not disappearing with insufflation (F1) [1] • Large varices occupying less than one-third of the lumen (F2) [2] • Large, coil-shaped varices occupying more than one-third of the lumen (F3) [3]
Colour (C) • White varices (Cw) • Blue varices (Cb) • Thrombosed varices (Cw-Th or Cb-Th)	Colour (C) • White varices (Cw) [+0.2085] • Blue varices (Cb) [-0.7188]	Colour (C) • White varices appearing like large folds of oesophageal mucosa (Cw) [0] • Varices that cannot be clearly assigned as Cw or Cb (Cbw) [1] • Dark blue varices, appearing cyanotic (Cb) [3]
Red colour sign (RC) • Red wale marking (RWM) • Cherry red spot (CRS) • Haematocystic spot (HCS) • RC(‐) • RC(+) • RC(++) • RC(++ +) • Telangiectasia [TE(+)]	Red colour sign (RC) • Both red wale marking and cherry-red spot were negative or mild (+) – *Group A *[+0.4046] • Both red wale marking and Red colour sign negative (RC-) [−0.2136] • Red u sign positive (RC+) [−0.2136] • Red wale marking (+), (++), (+++) [−0.8866] • Cherry red spot (‐), (+), (++), (+++) [+0.4468, +0.4468, +0.2429, +0.6727] • Haematocystic spot (HCS −/+) [+0.0516 / +0.6875] • Diffuse redness (DR -/+) and cherry-red spot were moderate (++) or severe (+++) – *Group B* [+0.0072 / +0.0550]	Red colour sign (RC) • Absent (0) • Present but no more than 10 lesions seen in oesophagus (+) [1] • More than 10 lesions are easily visible but not extensive (++) [2] • Extensive, covering all varices in the entire oesophagus (+++) [3]
Bleeding signs (B) During bleeding • Spurting • Oozing After haemostasis • Red plug • White plug	Adjunct finding • Oesophagitis [E(‐/+)] [+0.0723/−1.3090]	
Mucosal findings • Erosion (E) • Ulcer (UI) • Scar (S)			
**For Beppu classification: risk scoring end points**			
Total score and relation to bleeding	0 and -0.38 → 64.5% bleeders −0.38 and−1.14 → 90.2% bleeders ≤−1.14 → 100% bleeders		
**For Snady and Feinman’s classification: risk scoring end points**			
**Score**	**Grade**	**Percentage bleeding (on 2 years follow-up)**
1–3	Low	0%
4–7	Medium	11%
8–10	High	73%

Individual bleeding risk score is given in square brackets

In 1981, before the JSPH’s revised General Rules came into being, Beppu and colleagues had modified the original JSPH criteria for predicting variceal bleeding endoscopy or subsequent re-bleeding on emergency endoscopy (Table 2B) [34]. They assessed the risk of variceal bleeding in 172 patients through endoscopic findings, and found that the presence of red wale marks, cherry red spots of the red colour sign category and blue varices of fundamental colour category were the best predictors of variceal bleeding on follow-up, and that the forms of varices and their locations were not significant in predicting bleeds. In addition, in the Beppu classification, the red colour sign was divided into Groups A—in which red wale markings and cherry red spots indicated negative or mild—and B, in which both were moderate or severe. After identifying the high-risk factors for bleeding on endoscopy (colour blue, red wale marks +++, cherry red spot +++, haematocystic spot + and oesophagitis), the group applied discriminant analysis methodology to calculate a score that expressed the predictability of bleeding, relative to the respective categorical factors, utilizing the Quantification Method II proposed by Hiyashi [35]: the lower the score, the higher the discriminatory power that indicated bleeding, and *vice versa.*

Paquet’s classification of varices was published in 1982, based on a four-point Likert scale (a scale used to represent people’s attitudes to a topic or an evaluation methodology, providing quantitative value for any kind of subjective or objective item/process, with level of agreement/disagreement) [36], and many authors have since utilized this grading in their study cohort. In this classification, there are four grades of varices and their descriptors, as shown in Table 1C.

Westaby and colleagues proposed in 1984 a simple classification based on the luminal occupancy of oesophageal varices (Table 1D) [37], which has been endorsed by the British Society of Gastroenterology in its guidelines [38].

The Soehendra classification for oesophageal varices came into being in 1986 [39]. This classification came from descriptions of endotherapy (glue therapy) in patients with acute variceal bleeding and large varices (Table 1E).

In 1987 Pagliaro *et al.,* in order to evaluate the reliability of endoscopic assessment of variceal features in bleeding risk, studied 28 patients with liver cirrhosis and, based on the JSPH classification with the addition of a new item—oesophageal lumen occupancy—launched a semi-quantitative rating system for endoscopic findings. Known as the Italian Liver Cirrhosis Project Classification (ILCP), the authors used the following parameters to describe varices in their classification: location, size and occupancy [small straight varices (grade 1, small), enlarged tortuous varices occupying less than one-third of the lumen (grade 2, medium) and large coiled shaped varices occupying more than one-third of the lumen (grade 3, large)], blue tone, blue extension and red colour signs.

In 1988, the North Italian Endoscopic Club proposed characteristics, seen under endoscopy, that were predictive of bleeding, akin to the first such set of criteria proposed by Beppu *et al.* This classification was based on the sizes of varices, severity of red wale marks and the severity of underlying liver disease. Risk stratification was proposed for this classification, with cumulative scores for individual features totalled to define a risk class, as shown below in Table 3 [40, 41].

**Table 3.** Risk class prediction for variceal bleeding as per the North Italian Endoscopic Club Classification

Parameter	Risk score
Size of varices	
Small (< 25% lumen)	8.70
Medium (25 – 50%)	13.0
Large ( > 50%)	17.4
Red wale marks	
Absent	3.20
Mild	6.40
Moderate	9.60
Severe	12.80
Child Pugh class	
A	6.50
B	13.0
C	19.50
Risk class	Total score*
1	<20
2	20–25
3	25.1–30
4	30.1–35
5	35.1–40
6	>40

* The igher the score, the higher the risk class and the higher the chances of bleeding

In 1988, Snady and Feinman conceived a numerical grading system for varices based on size and other endoscopic criteria, yielding an aggregate variceal grade from 1 to 10 (Table 2C) [42]; high-grade varices were identified by a score of ≥8, with low grade scoring ≤7. Under this grading, 73% of patients with high-grade varices bled on follow-up of mean duration 26 months. The importance of this study was that it did away with the complex decimal system of bleeding risk prediction used in previous studies, in favour of a simplified scoring version that was easy to use.

Calès grading of oesophageal varices came in 1990, which roughly matched pre-existing grading systems in describing varices as small, medium and large (Table 1F) [43].

In 1991, a large multi-centre trial conducted by The Veterans Affairs Cooperative Variceal Sclerotherapy Group, led by Gregory *et al*.*,* described varices as small, medium and large, but with differences in the size distinctions [44]. In their study, small was <5 mm, medium was 5–9 mm and large >9 mm. This was similar to the classification given by Conn *et al*.** in 1967 but with a broader range in the sizes of varices.

In a consensus meeting held in 1990, at Baveno, Lake Maggiore, Italy, it was decided that a simple grading system be upheld for diagnosis of oesophageal varices. Hereafter, a system of small (<5 mm) and large (>5 mm) varices was adopted for ease of use by observers and management. Further Baveno guidelines followed this pattern of grading of oesophageal varices [45].

In 1996, the ILCP classification was modified with gastric features of portal hypertension such as the presence or absence of gastric varices and congestive gastropathy to produce a scoring system similar to the one proposed by Beppu and co-workers [46]. On Cox multivariate analysis, size, gastric varices and congestive gastropathy were the only independent predictors of bleeding and a prognostic index (PI) was developed, fulfilled by the formula PI = (size × 0.0395) + (congestive gastropathy × 0.878) + (gastric varices × 0.705), which was found to be superior to the NIEC index in predicting bleeding and for clear guidance in primary prophylaxis.

In 1997, the American College of Gastroenterology Practice Parameters Committee published its recommendations on variceal disease screening and management of acute variceal bleeding [47]. Even though there was no universal agreement on the grading of varices, as per research study methodology, the small, medium and large grades were adopted in this study.

Even though their work was published only as an abstract in *Gastrointestinal Endoscopy* in the year 2001, the CURE Hemostasis Research Group described what came to be known as the US System of Variceal Grading [48]. In their study, they described varices as small, medium, large and giant, with 2.3% of 251 patients studied having giant varices. In 2007, the American Association for Study of Liver Diseases published its own recommendations and approach to variceal disease [49]; it endorsed the Baveno guidelines in its study and recommended the simplest grading and classification for oesophageal varices. The year 2010 saw a revision of the Japanese General Rules [50]: the new rules added recommendations for diagnosis of portal hypertensive gastropathy and a new grading and classification based on endoscopic ultrasonography. In the previous version, gastric varices had been grouped alongside oesophageal varices, which were listed separately in the revised guidelines. Another modification was that of description of active bleeding during endoscopy—divided into gushing, spurting, oozing plus those covering recent haemostasis, such as red plug and white plug. The revised criteria were also the first to describe eradicated varices and recurrence of varices on endoscopy. Eradicated varices were described as F0RC0 (no form and red color signs), whereas recurrence of varices of varices were described as L_(location)_ F1RC_ [present/absent]_ (RWM). The description of residual varices on endotherapy (especially on sclerotherapy) was similar to normal descriptive terminologies. The endosonographic description of oesophageal varices provided in the revised General Rules included the following: -

- Oesophageal varices appear as hypoechoic or echo-free lumen in the submucosa
- Documentation of maximum minor axis diameter (D, in mm) of varices was made with variceal eradication, depicted as D (0)
- Recording further included that of the presence or absence of perforating veins (Pv+ or Pv-) with documentation of diameters of Pv.
- The small vessels adjacent to the muscularis externa of the oesophagus or partly invading the muscular wall of the oesophagus were described as peri-oesophageal veins (Peri-v+ or Peri-v-) and large vessels distal to the muscularis externa of the oesophagus (called paraesophageal; Para-v+ or Para-v-) were part of the description.
- The order of recording on EUS was [D, Pv, Peri-v, Para-v]
- In post-endotherapy patients, an additional recording of oesophageal wall hypertrophy (Hy) was to be made.

## Classification and grading of gastric varices

**Figure 3.**
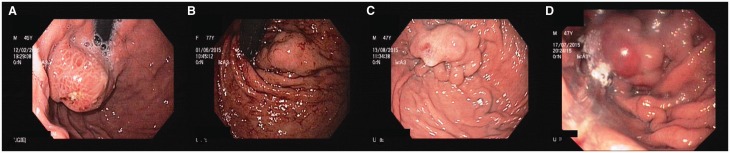
A) Large GOV2 (along greater curvature; also note the small ulcer on the surface, post glue therapy) with diffuse red colour signs; B) Large GOV1 (along lesser curvature of stomach); C) Large IGV1 with cherry red spot (form 3, tumorous); D) Large GOV1 with a haematocystic spot on the surface.

**Figure 4.**
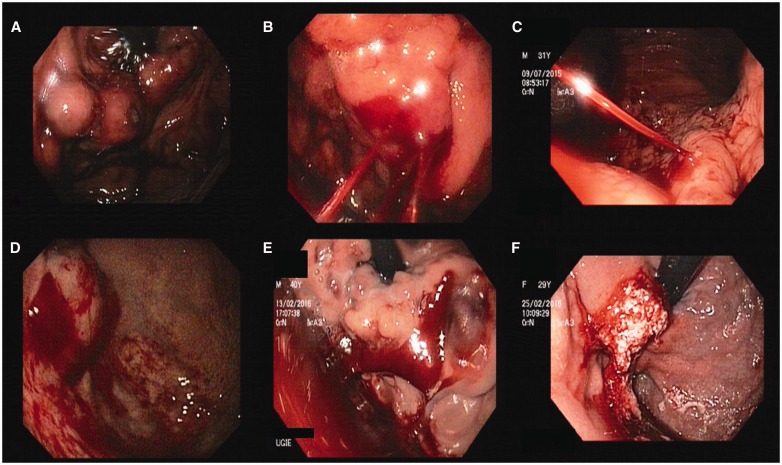
A) Bunch of GOV2 with active ooze from surface ulcer; B) Multiple site spurting from GOV2; C) End on view of a spurting GOV1 (along lesser curvature); D) Oozing IGV1 with adherent clot on the surface; E) Oozing GOV2 with adherent clots; F) Post-glue injection into a bleeding GOV1.

(Refer to figures 3 and 4

.) The formation of gastric varices in association with portal hypertension was first described by Stadelmann in 1913 [51]. Choi in 1983 classified gastric varices into ‘forms’, with F1 mild, F2 moderate and F3 severe type of gastric varices [52]. Further, a comprehensive classification for gastric varices was based on the presence of isolated splenic vein thrombosis (sinistral portal hypertension) or the presence of portal hypertension (cirrhotic or non-cirrhotic). Gastric varices due to left-sided portal hypertension usually arise from short gastric veins, from the hilum of the spleen running towards the greater curvature of the stomach (and not associated with large portosystemic shunts); they tend to be multiple and difficult to manage endoscopically, with high rates of recurrent bleeding. Hoskins and Johnson in 1988 gave the first full descriptive classification of gastric varices (Table 4A) [53]. Mathur *et al.* from India, in 1988 classified gastric varices into five types, shown in Table 4B [54]. Hashizume *et al*.** in 1990 classified gastric varices with reference to underlying vascular anatomy. In this classification, the varices were described under the headings of Form and Location and the presence or absence of red colour signs (Table 4C) [55].

**Table 4.** Important classifications of gastric varices

A. Hoskins and Johnson’s classification	C. Hashizume’s classification
**Type 1: **Included inferior extension of oesophageal varices across the squamo-columnar junction **Type 2: **Included gastric varices located in fundus, which appear to converge to cardia with oesophageal varices **Type 3**: Gastric varices in fundus or body in absence of oesophageal varices	**Form**: Tortuous (F1), nodular (F2) or tumorous (F3) **Location: **Anterior (La), posterior (Lp), lesser (Ll) and greater curvature cardiac (L) or fundic areas (Lf) **Colour**: Red (Cr) or colour white (Cw) and thin-walled focal redness on the varix as red colour spot (RCS)
B. Mathur’s classification	D. Sarin’s classification
**Type 1**: Oesophageal varices with lesser curvature varices. **Type 2**: Oesophageal varices with fundal varices (2a – Subcardiac and 2b – Diffuse fundal). **Type 3**: Isolated fundal varix (3a – Secondary to splenic vein thrombosis, 3b – Secondary to generalized portal hypertension). **Type 4**: Lesser curvature gastric varices with oesophageal varices with fundal varices. **Type 5**: Antral varices.	**Gastro-oesophageal varices Type 1**: Continuation of oesophageal varices into the lesser curvature (GOV1). **Gastro-oesophageal varices Type 2**: Oesophageal and fundal varices are present in continuity with the greater curvature (GOV2). **Isolated gastric varices Type 1**: Fundal varices are present in the cardia in the absence of oesophageal varices (IGV1). **Isolated gastric varices Type 2**: Fundal varices present in the stomach outside of cardio-fundal region or first part of duodenum (IGV2).

The most commonly followed—and Baveno consensus (1996)-endorsed—classification of gastric varices was provided by *Sarin et al*.** in 1992 [56]. In this classification, the importance of location was emphasized with reference to choice of therapy. The underlying vascular anatomy was not considered; hence alternative treatment plans cannot be made with this classification (Table 4D). Further classification of gastric varices was provided in 2002, with general division into cardiac and fundic varices [57]. Cardiac varices are those supplied by the cardiac branch of the left gastric vein, entering the stomach wall 2–3 cm from the gastro-oesophageal junction. Fundic varices receive their supply mostly from the short gastric vein but in some it is the posterior or left gastric vein. Arakawa *et al*. divided gastric varices into Type 1 (fundal—or localized—gastric varices, as per Iwase *et al*.*)* [58], in which a single dominant feeding channel arising from the splenic vein empties into the left renal vein through the gastric cardia and/or fundus, and Type 2 (diffuse gastric varices as per Iwase *et al*.*)* [58], in which the vessels empty into the left renal vein in the presence of multiple feeder collaterals. Apart from these, the JSPH General Rules modified the Hashizhume classification into gastric varices, isolated at fundus/fornix (Lg f), cardiac (Lg-c), or both (Lg c-f) and those at antrum (Lg-a) or body (Lg-b). The Italian Endoscopic Club classification described gastro-oesophageal varices as Type I gastric varices; and isolated gastric varices and ectopic gastric varices as Type II gastric varices.

## Conclusion

**Figure 5.**
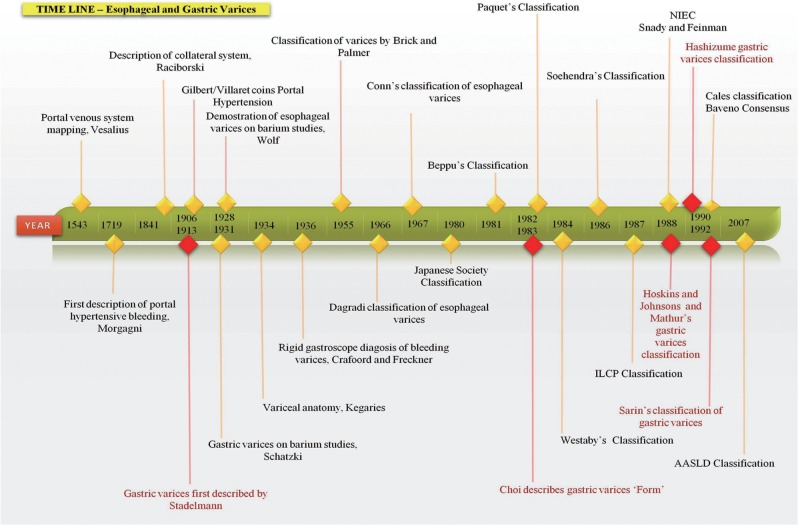
Timeline of historical events in classification and grading of oesophageal and gastric varices.

After much complexity and multiple classification systems, the classification of oesophageal varices that is most comprehensively followed and universally endorsed is currently the one accepted by the Baveno consensus, endorsed by the American Association for Study of Liver Diseases (AASLD), the European Association for Study of the Liver (EASL) and the Asian-Pacific Association for Study of the Liver (APASL) societies, which classifies varices (i) into small and large and (ii) the presence or absence of red colour signs. Even though many classifications for gastric varices exist, Sarin’s classification is the front runner because of ease of use and therapeutic indications; however, when it comes to alternative therapies for their treatment (such as transjugular intrahepatic portosystemic shunting or balloon retrograde trans-venous occlusion), with reference to anatomical descriptions, other classifications need to be addressed. A timeline of important events in the classification and grading of varices is shown in Figure 5

.

*Conflict of interest statement:* none declared.
